# Risk factors for onset of hypothermia in trauma victims: The HypoTraum study

**DOI:** 10.1186/cc11449

**Published:** 2012-07-31

**Authors:** Frédéric Lapostolle, Jean Luc Sebbah, James Couvreur, François Xavier Koch, Dominique Savary, Karim Tazarourte, Gerald Egman, Lynda Mzabi, Michel Galinski, Frédéric Adnet

**Affiliations:** 1 AP-HP, Urgences - SAMU 93, Unité recherche-enseignement-qualité, Hôpital Avicenne, 125, rue de Stalingrad, F-93000 Bobigny, France; 2 Université Paris 13, Sorbonne Paris Cité, EA 3509, F-93000 Bobigny, France; 3 SMUR, Centre Hospitalier de Gonesse, 25 rue Bernard Février, 95500 Gonesse, France; 4 SAMU 83, Centre Hospitalier Intercommunal, 54, rue Henri Sainte Claire Deville BP 1412, 83056 Toulon cedex, France; 5 Pôle Urgence-SAMU-SMUR, Hôpital Michallon, Boulevard de la Chantourne, 38701 La Tronche, France; 6 SAMU 74, avenue de l'hôpital, 74374 Pringy, France; 7 Pôle urgence-réanimation-SAMU 77, Centre Hospitalier Marc Jacquet, 11 Rue Freteau de Peny, 77000 Melun, France; 8 SAMU 973, Centre Hospitalier Andrée Rosemon, 97306 Cayenne, Guyane Française, France

## Abstract

**Introduction:**

Hypothermia is common in trauma victims and is associated with an increase in mortality. Its causes are not well understood. Our objective was to identify the factors influencing the onset of hypothermia during pre-hospital care of trauma victims.

**Methods:**

This was a multicenter, prospective, open, observational study in a pre-hospital setting.

The subjects were trauma victims, over 18 years old, receiving care from emergency medical services (EMS) and transported to hospital in a medically staffed mobile unit.

Study variables included: demographics and morphological traits, nature and circumstances of the accident, victim's presentation (trapped, seated or lying down, on the ground, unclothed, wet or covered by a blanket), environmental conditions (wind, rain, ground temperature and air temperature on site and in the mobile unit), clinical factors, Revised Trauma Score (RTS), tympanic temperature, care provided (including warming, drugs administered, infusion fluid temperature and volume), and EMS and hospital arrival times.

**Results:**

A total of 448 patients were included. Hypothermia (<35°C) on hospital arrival was present in 64/448 patients (14%). Significant factors associated with the absence of hypothermia in a multivariate analysis were no intubation: Odds Ratio: 4.23 (95% confidence interval 1.62 to 1.02); RTS: 1.68 (1.29 to 2.20); mobile unit temperature: 1.20 (1.04 to 1.38); infusion fluid temperature: 1.17 (1.05 to 1.30); patient not unclothed: 0.40 (0.18 to 0.90); and no head injury: 0.36 (0.16 to 0.83).

**Conclusions:**

The key risk factor for the onset of hypothermia was the severity of injury but environmental conditions and the medical care provided by EMS were also significant factors. Changes in practice could help reduce the impact of factors such as infusion fluid temperature and mobile unit temperature.

## Introduction

Trauma victims often suffer from hypothermia on arrival at hospital and, even when the hypothermia is moderate, it can be associated with a poorer prognosis and an increase in mortality rate [1-8]. Early diagnosis of hypothermia is thus essential [3]. However, although the mechanisms of the deleterious effects of hypothermia are well known, its causes are not clear. Most published data on hypothermia victims are hospital registry data or data from retrospective studies [3-10]. Data from a pre-hospital setting are scarce.

A possible cause of hypothermia is the severity of the injury [1,3-8,11]. Both severe head injury and hypovolemic shock affect body temperature regulation. However, the contribution of other factors and their potential interactions are not known. They include, to list but a few, weather conditions (cold, wind, rain, and length of exposure) and pre-hospital care by emergency medical services (EMS). Although protecting the victim from the cold or warming them, whether passively or actively, might have a positive effect on body temperature, treatment such as vascular filling might impact negatively [12].

The aim of our study was to identify the risk factors associated with the onset of hypothermia when EMS provided pre-hospital care to trauma victims. Better knowledge of these factors might help prevent hypothermia and improve prognosis.

## Methods

### Study design and setting

This was a prospective, multicenter, open, observational study carried out by the mobile EMS of eight French hospitals between 1 January 2004 and 10 November 2007. In France, the SAMU (Service d'Aide Médicale Urgente), is called for the management of patients in pre-hospital settings. The most appropriate response is decided by an emergency physician, 'the SAMU dispatcher'. When required, he can send a medical team to manage critical patients. This squad is composed of an emergency physician, a nurse specialized in critical care and a driver with basic live support training. Ambulances are equipped with intensive care facilities, including drugs for anesthesiology and catecholamines, biology facilities, and ultrasound devices [13,14]. For more details regarding the organization of pre-hospital emergency care in France, see [15].

### Inclusion and exclusion criteria

We included all trauma victims over 18 years old who received pre-hospital care from EMS and who were transported to hospital in a medically equipped mobile unit. We excluded patients who were not transported in a mobile unit and those with bilateral aural bleeding preventing continuous monitoring of tympanic temperature.

### Study variables

We recorded demographics and morphological traits (age, sex, body weight and height of the victim), the nature and circumstances of the accident (date, time, place), the victim's presentation on EMS arrival (trapped or not, seated or lying down, on the ground, unclothed, wet, or covered by a blanket), environmental conditions, clinical factors, and care provided. Environmental conditions included air (indoors or outdoors) and ground temperature, wind speed (maximum and mean), and rain at the site of the accident. The conditions were considered windy if maximum wind speed was >15 km/hr or mean speed was >10 km/hr. The conditions also included air temperature in the mobile unit and the temperature at the emergency department's entrance hall. Clinical factors included: the site and nature of the lesions (fracture, wound, contusion), heart rate, the Revised Trauma Score (RTS; Glasgow Coma Score (GCS) + systolic blood pressure + respiratory rate), oxygen saturation (and oxygen delivery), and tympanic temperature. Heart rate, systolic blood pressure and oxygen saturation were automatically measured using multiparametric monitoring devices. The following aspects of care were recorded, whenever applicable: vascular filling (infusion fluid temperature and volume), catecholamine and morphine administration, any other drugs (with doses), orotracheal intubation, and warming of the victim (passive or active). We also recorded the times from the accident to EMS arrival and from EMS arrival to hospital admission.

### Temperature and wind measurements

Body temperature was monitored continuously throughout patient care using a tympanic thermometer (Métraux®, Crissier, Switzerland) which measures temperature by infrared (IR) measurement [16]. Infusion fluid temperature was measured using a previously validated instrument [17]. We demonstrated that IR measurement was strongly correlated with temperature sensor measurement, the gold standard method [17]. Infusion fluid, air and ground temperatures were measured using a non-contact IR thermometer (TN1 Nonfumo flue systems^®^, High Wycombe, UK). Body, air and ground temperature were measured when the victim was first examined. Body and mobile unit air temperature were then measured when the victim was placed in the mobile unit, on departure of the mobile unit, and on arrival at the hospital door. Furthermore,, body temperature was recorded every 15 minutes.

Wind speed (maximum and mean) was measured by exposing an anemometer (La Crosse Technology^®^, Geispolsheim, France) to the wind for at least five seconds on first examination of the victim.

### Primary endpoint

The primary endpoint was hypothermia on arrival at the hospital. It was defined as a body temperature of <35°C [3,5-7,9,10]. Trauma victims with and without hypothermia were compared for each of the above listed study variables and for severity of injury as given by clinical criteria and RTS.

### Statistical analysis

Results were expressed as proportions of victims or medians with 25 to 75 percentiles. Quantitative data were compared using the Mann-Whitney test and qualitative data using the Chi-square test. *P *values of 0.05 or less were considered significant. Variables with a *P *value of <0.2 were entered into a multivariate logistic regression model (Statview 5.0, SAS Institute, Cary, NC, USA). Odds ratios (OR) were calculated.

The study was approved by the local Ethics Committee (Committee for the Protection of Persons - CPP Ile de France, Hôpital Robert Ballanger, Aulnay-sous-bois, France). Because patient care was not altered in any way, no informed consent signature was required under French law.

## Results

**Figure 1:**
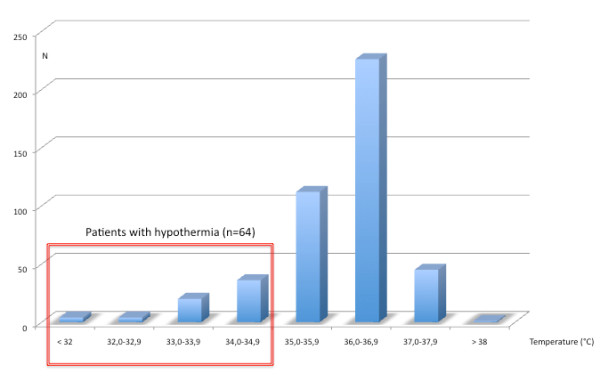
**Temperature distribution in the 448 trauma victims on arrival at hospital**.

We included 461 patients. Body temperature measurements on arrival at hospital were available for 448 patients (97%). The distribution is shown in Figure 1. Hypothermia (body temperature <35°C) was present in 64 of these 448 patients (14%). The proportion of patients with hypothermia and the median air temperature are given in Table 1 for each participating hospital.

**Table 1:** Patients with hypothermia on arrival at the participating hospitals.

Hôpital	Inclusions Number	Patients with hypothermia on arrival Number (%)	Median air temperature °CI (25 to 75 percentiles)
Bobigny	251	31 (12)	17.5 (10.8-23.0)
Gonesse	99	8 (8)	16.7 (9.0-21.6)
Brest	39	15 (41)	14.4 (11.0-19.2)
Grenoble	22	4 (18)	6.2 (-2.0-20.0)
Annecy	14	5 (36)	14.8 (12.4-20.3)
Voiron	11	0 (0)	16.1 (8.4-20.4)
Melun	10	1 (10)	23.5 (21.0-25.4)
Cayenne	2	0 (0)	26.0 (25.4-29.7)
Total	448	64 (14)	17.0 (10.3-22.3)

CI, confidence interval.

Patients with and without hypothermia on hospital arrival are compared in Table 2 (demographics and morphological traits, circumstances of the accident, and environmental conditions) and Table 3 (presentation of victim and on-site clinical assessment and care). There was no significant difference in the demographics of the two groups. All the factors with a *P *value of <0.2 in the univariate analyses of Tables 2 and 3 were entered into the multivariate analysis except for the following: ground temperature, mobile unit temperature on departure from accident site, GCS, and systolic blood pressure as these were directly related to either the air temperature or RTS. The independent factors associated with no hypothermia on arrival at hospital in the multivariate analysis are given in Table 4. The most significant was the severity of the injury (RTS) but care procedures (intubation and vascular filling) also proved to be highly significant.

**Table 2:** Patient demographics, circumstances of accident, and environmental conditions.

	Patients with hypothermia on arrival at hospital	Patients without hypothermia on arrival at hospital	*P*
**Demographics and morphological traits**			

Age (yearrs)	36 (23-53)	33 (23-44)	0.2
Male - Number (%)	49 (77)	275 (72)	0.7
Body weight (kg)	72 (60-80)	72 (65-80)	0.6
Height (cm)	172 (169-180)	174 (166-180)	0.9
Body Mass Index	24.2 (21.2 - 27.7)	24.5 (21.8 - 26.9)	0.5

**Circumstances of accident - Number (%)**			

Road accident	32 (50)	220 (57)	0.06
Fall	23 (36)	117 (30)	
Weapon (gun or knife)	2 (3)	18 (5)	
Other	7 (11)	29 (8)	

**Environmental conditions**			

Daytime - Number (%)	37 (58)	262 (68)	0.5
Winter - Number (%)	20 (32)	110 (29)	0.09
Spring - Number (%)	14 (22)	91 (24)	
Summer - Number (%)	12 (19)	105 (27)	
Autumn - Number (%)	18 (28)	78 (20)	
Indoors - Number (%)	10 (16)	72 (19)	0.6
Air temperature (°C)	11.5 (6.7-16.4)	17.9 (11.0-23.0)	0.0001
Ground temperature (°C)	11.4 (7.1-17.1)	18.1 (11.1-22.9)	<0.0001
Windy - Number (%)	8 (13)	28 (7)	0.2
Rain - Number (%)	10 (16)	37 (9)	0.3

Values are given as medians with 25 to 75 percentiles.

**Table 3:** Patient presentation and on-site clinical assessment and care.

	Patients with hypothermia on arrival at hospital	Patients without hypothermia on arrival at hospital	*P*
Trauma victims - Number (%)	62 (13)	399 (87)	

**Presentation of victim**			

Trapped - Number (%)	7 (11)	51 (13)	0.6
Seated - Number (%)	6 (9)	61 (16)	0.2
Lying - Number (%)	58 (91)	319 (83)	
On the ground - Number (%)	51 (80)	215 (56)	0.01
Unclothed - Number (%)	25 (39)	117 (30)	0.2
Covered by a blanket - Number (%)	33 (53)	194 (49)	0.8
Wet - Number (%)	14 (22)	34 (9)	0.01

**Clinical examination**			

Head injury - Number (%)	45 (70)	165 (43)	0.0001
Chest injury - Number (%)	24 (37)	112 (29)	0.2
Abdominal injury - Number (%)	7 (11)	51 (13)	0.8
Hip injury - Number (%)	10 (16)	66 (17)	1
Limb injury - Number (%)	33 (52)	149 (39)	0.04
Glasgow Coma Score (GCS)	12 (5-15)	15 (15-15)	0.0001
Systolic blood pressure (mmHg)	118 (74-139)	125 (110-140)	0.01
Heart rate (bpm)	88 (70-110)	88 (75-100)	0.5
Respiratory rate (breaths/min)	18 (15-24)	18 (16-22)	0.6
Pulse oxymetry (SpO_2_)	98 (96-100)	99 (97100)	0.2
Revised Trauma Score (RTS)	10 (8-11)	11 (11-11)	<0.0001
Initial tympanic temperature (°C)	34.2 (33.2-34.8)	35.7 (35.1-36.4)	<0.0001

**On-site management**			

Time from accident to EMS arrival (min)	30 (22-43)	30 (24-45)	0.8
Mobile unit temp on arrival (°C)	21.7 (19.6-23.0)	22.0 (20.0-25.2)	<0.0001
Lowest mobile unit temperature (°C)	19.8 (18.0-22.0)	21.7 (19.7-24.9)	<0.0001
Vascular filling volume (ml)	510 (300-1250)	300 (210-500)	<0.0001
Infusion fluid temperature (°C)	19.5 (17.7-21.0)	22.0 (19.2-24.9)	<0.0001
Catecholamine - Number (%)	9 (14)	7 (2)	<0.0001
Orotracheal intubation - Number (%)	32 (50)	25 (7)	<0.0001
Morphine - Number (%)	37 (58)	223 (58)	1
Warming - Number (%)^a^	56 (87)	284 (74)	0.03
Time from EMS arrival to hospital admission (min)	65 (53-90)	60 (45-75)	0.01

^a^Active in 14 patients. Values are given as medians with 25-75 percentiles. EMS. Emergency medical services.

**Table 4:** Independent factors for absence of hypothermia in a multivariate analysis.

Factors for absence of hypothermia	Odds Ratio (95% CI)	*P*
No intubation	4.23 (1.61-11.02)	0.003
Revised Trauma Score	1.68 (1.29-2.20)	0.0001
Mobile unit temperature on arrival on site	1.20 (1.04-1.38)	0.01
Infusion fluid temperature	1.17 (1.05-1.30)	0.003
Patient not unclothed	0.40 (0.18-0.90)	0.03
No head injury	0.36 (0.16-0.83)	0.01

CI, confidence interval.

## Discussion

The prevalence of hypothermia in trauma victims on arrival at hospital was high (14%) and was associated with several risk factors. The most significant factor was the severity of the injury as given by the RTS. The relationship between hypothermia and severity of injury is known [10]. Blood loss and spine or head injury impair body temperature regulation, even if not always immediately. Intubation was also a significant risk factor. It was no doubt a sign of the severity of the injury [8]. In France, it is a common procedure in a pre-hospital setting that is not reserved for severe head injuries only [15,18]. Patients with respiratory distress and severe multiple trauma often require ventilation as well [13]. Head injury, RTS, and intubation were, all three, severity criteria independently associated with hypothermia.

To our knowledge, infusion fluid temperature was shown, for the first time, to be a significant risk factor for the onset of hypothermia [12,19]. The temperature of the fluid infused on resuscitation of 75% of the patients with hypothermia on arrival at hospital was below 21°C and close to air temperature. Infusion temperature was a more important risk factor than infusion volume, maybe partly because of the small infusion volumes used. Another significant factor was mobile unit temperature. In order to minimize hypothermia in trauma victims, we therefore recommend that infusion fluid temperature be controlled, that small infusion volumes be used for resuscitation, that the mobile unit be heated, and that trauma victims preferably remain clothed. It is common practice among first aid workers to undress the victim before EMS arrival for a complete clinical examination.

Earlier studies have suggested that the season of the year might play a role in hypothermia [5,10]. Our multivariate analysis did not identify air and ground temperatures at the site of the accident as independent risk factors. Prevalence of hypothermia was high even when air temperature was mild. However, weather conditions probably exerted an indirect influence by affecting mobile unit and infusion fluid temperature, which are perforce somewhat related.

The relative contribution of each of the three most significant risk factors (severity of injury, infusion fluid temperature, and mobile unit temperature) in the onset of hypothermia no doubt varied according to circumstance and weather conditions; the circumstances of the accident, and pre-hospital care may have masked other influential factors [7]. Being trapped is not necessarily associated with severity of injury and was not a significant risk factor. It can protect against the cold and rain but may prolong out-of-hospital care. There were too few trapped victims in our study for an analysis of time spent being trapped. In general, however, times taken, which often depend on the severity of the injury and on resuscitation procedures, did not seem to influence onset of hypothermia. The median time from the accident to clinical examination by EMS was 30 minutes, and from the examination to arrival at hospital was 65 minutes. Warming of the victim was not associated with a lower incidence of hypothermia but active warming was seldom practiced. Warming did not compensate for the effect of the victim being unclothed.

Optimal patient management could contribute to limit heat loss or even to increase the patient's temperature when required. Undressing patients should be avoided. Mobile unit temperature and fluid infusion temperature were independently associated with hypothermia. They should be routinely measured. Adapted tools are available [17]. They should be warmed if necessary. Mobile intensive care units are equipped with refrigerators. They should probably also be equipped with a warming system to allow 'body temperature' fluid infusion, especially in severely injured patients. A patient's re-warming system should contribute to warm fluid infusion.

A strong point of our study was repetitive, prospective, and consistent measurement of temperature. Temperature measurements were easy to perform and instantaneous, whether for non-invasive continuous monitoring of body temperature or measurement of air and infusion fluid temperature [16,17]. However, our results cannot be generalized to all trauma victims as the most severe cases, in whom on-site resuscitation failed, and the least severe cases were not transported in the medically equipped mobile unit. Hypothermia is an unlikely prognostic factor for outcome in these cases. Although the severity of injury was moderate in our study, nearly a third of the victims had an initial body temperature below 35°C. A limitation of our study is that we did not include outcomes but the relationship between hypothermia and death is now well established [2,4,6,8,9,20].

## Conclusions

Routine temperature measurements should help improve the care of trauma victims. When providing early care, EMS should always look for hypothermia. The severity of injury, mobile unit temperature, and medical interventions were risk factors associated with hypothermia on the victim's arrival at hospital. Mobile unit and infusion fluid temperature should be measured and increased if necessary. Unclothing of patients should be avoided. A prospective study is needed to assess the impact of warming of the victim on morbidity and mortality.

## Key messages

• Routine temperature measurements should help improve the care of trauma victims.

• The severity of injury was associated with hypothermia on the victim's arrival at hospital.

• Mobile unit and infusion fluid temperature should be measured and increased if necessary.

• Unclothing of patients should be avoided.

## Abbreviations

EMS: emergency medical services; GCS: Glasgow Coma Score; IR: infrared; OR: odds ratios; RTS: Revised Trauma Score.

## Competing interests

The authors declare that they have no competing interests.

## Authors' contributions

All authors have made substantial contributions to the manuscript: FA and FL designed the study. JLS, JC, FXD, DS, KT and GE acquired data. FL, ML and MG analyzed and interpreted the data, and FL drafted the manuscript. All authors were involved in revising the manuscript critically and approved the final version.
